# Trophic Enrichment Factors of Carbon and Nitrogen Isotopic Ratios (Δ^13^C and Δ^15^N) in Four Marine Ciliates

**DOI:** 10.3389/fmicb.2021.721157

**Published:** 2021-09-23

**Authors:** Jun Young Park, Jae-Ho Jung, Jung Hyun Kwak, Heum Gi Park, Chang-Keun Kang, Hyun Je Park

**Affiliations:** ^1^Department of Marine Bioscience, Gangneung-Wonju National University, Gangneung, South Korea; ^2^Department of Biology, Gangneung-Wonju National University, Gangneung, South Korea; ^3^Jeju Fisheries Research Institute, National Institute of Fisheries Science, Jeju, South Korea; ^4^School of Earth Sciences and Environmental Engineering, Gwangju Institute of Science and Technology, Gwangju, South Korea

**Keywords:** isotopic fractionation, Δ^13^C, Δ^15^N, protozoa, stable isotopes, turnover rates, feeding experiment

## Abstract

Understanding the magnitude and causes of isotopic fractionation between organisms and their dietary resources is crucial for gaining knowledge on stable isotope ecology. However, little is known regarding the diet-tissue fractionation values of marine ciliates, which play a critical role in the reconstruction of microbial food webs. In the present study, we conducted experiments on two benthic (*Pseudokeronopsis pararubra* and *Protocruzia labiata*) and two pelagic (*Strombidium sulcatum* and *Uronemella filificum*) marine ciliates, where they were fed with isotopically constant foods (*Chaetoceros calcitrans* and *Isochrysis galbana*) under laboratory culture conditions to determine their carbon and nitrogen isotopic fractionation values (Δ^13^C and Δ^15^N). The stable isotope values (δ^13^C and δ^15^N) of ciliates for all experiments rapidly increased after the initial feeding, with half-lives ranging from 6.1 to 23.0h for δ^13^C and from 3.1 to 24.9h for δ^15^N. The Δ^13^C and Δ^15^N for all ciliates represented significantly positive enrichments, with overall mean fractionations of 0.6±0.2 and 1.2±0.4, respectively. Irrespective of the dietary type, both Δ^13^C and Δ^15^N were very similar for the same ciliate species. These results suggest that Δ^13^C and Δ^15^N for marine ciliates are similar to those found in common marine organisms with very little food-dependent variation. Overall, quantifying the specific isotopic fractionation of marine ciliates is expected to provide fundamental information on the trophic transfer of carbon, nitrogen, and energy flow through the microbial pathway in marine ecosystems.

## Introduction

Stable isotope ratios of carbon and nitrogen have been used to identify trophic pathways of organic matter and trophic relationships within animal communities, and the isotopic ratios of an organism’s tissues reflect those of its dietary sources (Fry and Sherr, 1984; Peterson and Fry, 1987; Michener and Schell, 1994; Layman et al., 2012). Typical enrichment of heavier isotopes in animal tissues occurs during the course of physiological metabolism, in the range of ≤1‰ for carbon (DeNiro and Epstein, 1978; Fry and Sherr, 1984) and 2–4‰ for nitrogen (Vander Zanden and Rasmussen, 2001; Post, 2002). The smaller fractionation of δ^13^C makes it useful for identifying the origins of dietary sources for organisms with distinctly different δ^13^C signatures among different organic matter sources, whereas δ^15^N is better suited for estimating their trophic levels (Peterson and Fry, 1987). However, estimating the contribution of dietary sources to the consumers’ production using stable isotopes can cause uncertainties owing to a variety of biotic and abiotic factors, there being too many sources of organic matter, isotopic discrimination between consumers and resources, and physiological conditions (Boecklen et al., 2011). Above all, estimates of trophic discrimination between consumers and resources (expressed by Δ^13^C for carbon and Δ^15^N for nitrogen) are considerably variable, which may result in various errors in the determination of resource contribution and trophic level (McCutchan et al., 2003). The application of actual trophic discrimination from stable isotope ratios will improve the accuracy of estimates of the contribution of dietary sources to the trophic level of consumers within diverse natural ecosystems (Post, 2002; McCutchan et al., 2003). Thus, understanding the magnitude and causes of isotopic discrimination between consumers and resources is crucial for understanding trophic ecology using stable isotope tracers.

Ciliates are one of the major functional groups in marine ecosystems (Pierce and Turner, 1992; Löder et al., 2011; Xu et al., 2018). They play important roles as intermediators in the pathways of material and energy flows from pico- and nanoplanktonic producers to higher trophic levels in marine ecosystems through the microbial food web (Montagnes et al., 2010; Zingel et al., 2019). Microzooplankton, including ciliates, is known to consume 60–75% of primary production and most of the bacterial secondary production in the oceans (Sherr and Sherr, 1994; Calbet and Landry, 2004). Several studies have focused on the importance of ciliates as a food source for mesozooplankton to establish the relevance of the trophic link in oceanic biogeochemical cycles on a global scale (Fessenden and Cowles, 1994; Calbet and Saiz, 2005). Given the magnitude of carbon flux from ciliates in marine food webs for carbon budget estimates, a comprehensive understanding of the phytoplankton-ciliate-mesozooplankton trophic pathways is required. However, the strength and variability of trophic linkages are difficult to quantify in complex natural systems, thus rendering the comparison of ecosystems unviable (Gutiérrez-Rodríguez et al., 2014). To determine the trophic pathways of ciliates within complex microbial food chains, tracking the dietary utilization of ciliates in food webs and demonstrating their nutritional contribution to higher trophic levels should be considered in a rigorous trophic analysis. Recently, stable isotope analysis has been applied to resolve trophic connections between phytoplankton, heterotrophic protists, and mesozooplankton, quantifying the energy flows of carbon and nitrogen through complex microbial food webs (Gutiérrez-Rodríguez et al., 2014; Décima et al., 2017). The isotopic discrimination values of specific organisms are necessary to determine the dietary sources that contribute to their nutrition and trophic levels in the food web (Post, 2002). Although isotopic values can provide quantitative and qualitative information regarding the transfer of material and energy in marine food webs, little is known about the diet-tissue fractionation values of marine ciliates that can be used as a critical value to reconstruct dietary sources and food web structures.

In the present study, we conducted feeding experiments on benthic and pelagic marine ciliates in laboratory cultures to determine the carbon and nitrogen isotopic fractionation values between diet and consumers. Such feeding experiments are indispensable for isolating and collecting sufficient amounts of ciliate samples for isotope analyses. During the experiments, the ciliates were fed with an isotopically constant food source over a period of time to precisely estimate isotopic fractionation. Furthermore, we also investigated whether the different types of diets for ciliates would influence their carbon and nitrogen isotopic fractionation through the isotope analysis of the ciliate populations raised on each type of diet.

## Materials and Methods

### Cultures and Feeding Experiments

**Figure 1:**
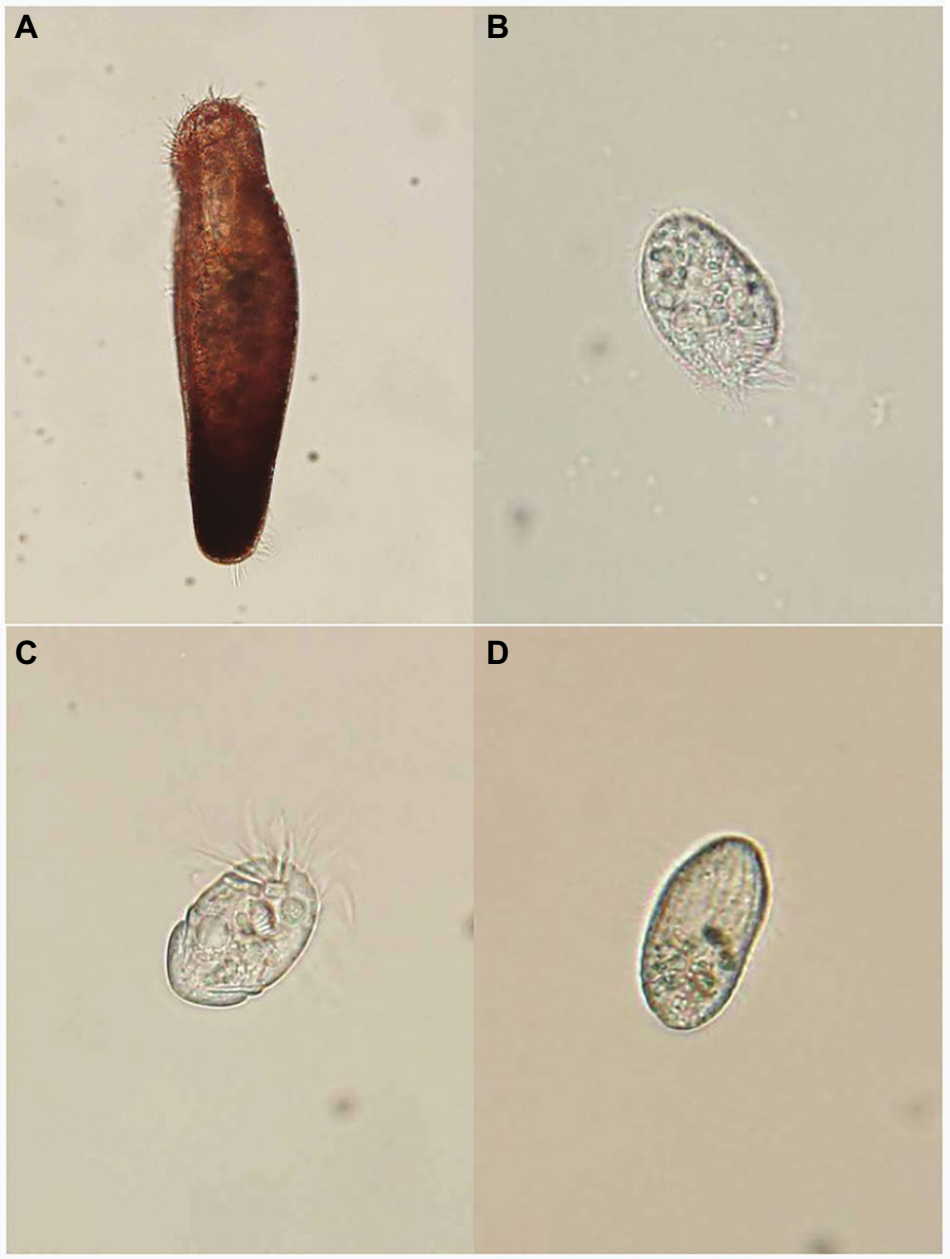
Specimens of four ciliates: **(A)** *Pseudokeronopsis pararubra*, **(B)** *Protocruzia labiata*, **(C)** *Strombidium sulcatum*, and **(D)** *Uronemella filificum*.

Two benthic (*Pseudokeronopsis pararubra* and *Protocruzia labiata*) and two pelagic (*Strombidium sulcatum* and *Uronemella filificum*) ciliates were obtained from the marine ciliate resource bank of Korea, Gangneung-Wonju National University, Gangneung, Korea (Figure 1). All ciliate organisms were cultured in f/2 seawater media at 20°C under a 14:10 light:dark (L:D) cycle with cool-white fluorescent lamps (irradiance 60μmol photons m^2^ s^−1^). The stock cultures were acclimated in cell culture flasks containing 300ml fresh f/2-Si seawater media and wheat grains (*Triticum aestivum* L.) as food to achieve initial cell concentrations.

Feeding experiments lasted 8days in 500ml transparent polycarbonate Nalgene bottles, which were considered sufficient for reaching isotope equilibrium. The experimental conditions were maintained in all the bottles and checked every 12h (temperature, average 15.3±0.6°C; salinity, average 25.0±0.3; dissolved oxygen, average 5.3±0.4mgL^−1^). Living microalgae for feeding experiments were supplied every day (average concentration 10×10^3^±480 cells mL^−1^) to the ciliate bottles. The diatom and haptophyte species, *Chaetoceros calcitrans* and *Isochrysis galbana*, respectively, which are dietary items of ciliates, were pure-cultured in rotating cell culture flasks in a low-temperature incubator until a uniform exponential growth phase. Controls of the four ciliates cultured with wheat grains were also maintained under the same experimental conditions. All treatment and control cultures were performed in duplicates and incubated for the experiments. The 3–5 ciliate samples from each bottle were collected at 0, 1, 4, 8, 12, 24, 48, 96, and 192h for analyses of cell abundance and carbon and nitrogen stable isotopes. The settled detrital materials were removed every day by careful pipetting to prevent the proliferation of bacterial populations and particle sedimentation.

At each collection, for the carbon and nitrogen stable isotope analyses, 20–40ml subsamples (presented from a preliminary experiment) from the treatment and control culture bottles were pre-filtered through 40μm Nitex sieves to remove any microalgae-derived particles, and then, the remaining ciliates were filtered onto pre-combusted (450°C, 4h) Whatman GF/F filters. Two diatom samples were obtained by filtering the culture water (approximately 10ml) through pre-combusted Whatman GF/F filters. All filter samples were oven-dried at 50°C for 48h, wrapped in aluminum foil, and stored at −40°C until isotope analysis.

### Carbon and Nitrogen Stable Isotope Analyses

To measure the stable isotope ratios, the filter samples were sealed in a tin disk. The sealed samples were analyzed using an elemental analyzer (Vario MICRO Cube, Elementar, Hanau, Germany) coupled to a continuous-flow isotope-ratio mass spectrometer (*CF*-IRMS; Isoprime 100; Isoprime Ltd., Cheadle Hulme, United Kingdom). The samples were oxidized at a high temperature (1,150°C) in the elemental analyzer, and the resultant CO_2_ and N_2_ gases were transferred into the *CF*-IRMS using helium as a carrier gas. The stable isotope ratios are expressed as δ notation relative to Pee Dee Belemnite for carbon and atmospheric air for nitrogen, using the equation: δ (‰)=[(*R*_sample_/*R*_standard_)−1]×10^3^, where R is the ^13^C/^12^C or ^15^N/^14^N ratio. The international standard reference materials, sucrose (ANU C_12_H_22_O_11_, δ^13^C=−10.47±0.13‰, NIST, Gaithersburg, MD, United States), and ammonium sulfate [(NH_4_)_2_SO_4_, δ^15^N=−1.8±0.1‰, NIST] were used for calibrating carbon and nitrogen, respectively, after analyzing every 10 unknown samples. The analytical precisions, based on repeated analyses of acetanilide (Thermo Scientific) as an internal standard, were within 0.07‰ for δ^13^C and 0.11‰ for δ^15^N.

### Carbon and Nitrogen Isotope Fractionations and Turnover Times

The trophic enrichment factors (TEFs, Δ) were calculated using the equation (Fry, 2006): Δ=δ_c_−δ_f_, where δ_c_ and δ_f_ refer to the carbon and nitrogen isotope ratios of the consumer species and food source at isotopic equilibrium, respectively. The equilibrated times were estimated for each feeding experiment by fitting the exponential decay model to the time of the asymptotic isotope values of consumers on the food source. To compare the variability in isotope fractionation between consumers and diets and among consumers, the standard deviations from the isotope mean values after equilibration were calculated.

To estimate the turnover time of carbon and nitrogen in the algal species, *C. calcitrans* and *I. galbana*, we fitted the isotope data to an exponential rise to a maximum equation, y=a+be^ct^, using SigmaPlot for Windows (version 14.0; Bosley et al., 2002). Here, y represents the δ^13^C or δ^15^N values of the two diets at the time, a and b are the regression coefficients (parameters) that provide the best fit between the equation and the data, c is the turnover rate of carbon or nitrogen in the two diets, and t is the time (h) of the diet switch. We calculated the half-life of carbon and nitrogen in the tissue using the equation half-life=ln(0.5)/c (Hobson and Clark, 1992).

### Statistical Analysis

All data were examined for normality using the Shapiro–Wilk test and homogeneity of variance with the Levene’s test prior to statistical analysis. Significant differences in the δ^13^C and δ^15^N values of microalgal diets and ciliates among culturing times during the feeding experiments, and the Δ^13^C and Δ^15^N values among culturing ciliates were analyzed using a one-way ANOVA. Subsequently, a Tukey’s multiple comparison *post-hoc* test was used to evaluate differences among variables. The significance level among treatments was α=0.05. All statistical analyses were performed using IBM SPSS Statistics software (version 23.0, IBM, Armonk, NJ, United States).

## Results

### Carbon and Nitrogen Stable Isotope Ratios and Turnover Times

The isotopic ratios of the two dietary microalgae (*C. calcitrans* and *I. galbana*) remained constant throughout the culture period (Table 1). There were no significant differences in the δ^13^C and δ^15^N values of both *C. calcitrans* and *I. galbana* at various sampling times, showing overall mean values of −14.5±0.3‰ and 9.9±0.2‰ (ANOVA, *F*_8,30_=1.04, *p*=0.435 for δ^13^C and *F*_8,30_=0.89, *p*=0.539 for δ^15^N) and−15.7±0.2‰ and 4.7±0.2‰ (ANOVA, *F*_8,28_=0.33, *p*=0.947 for δ^13^C and *F*_8,28_=1.39, *p*=0.260 for δ^15^N), respectively.

**Table 1:** Mean δ^13^C and δ^15^N values of food sources (*Chaetoceros calcitrans* and *Isochrysis galbana*) for marine ciliates during the entire experiment.

Time(h)	*Chaetoceros calcitrans*					*Isochrysis galbana*				
		δ^13^C		δ^15^N			δ^13^C		δ^15^N	
	n	Mean	*SD*	Mean	*SD*	n	Mean	*SD*	Mean	*SD*
0	5	−14.3	0.3	9.9	0.2	5	−15.6	0.2	4.6	0.2
1	3	−14.6	0.1	9.8	0.1	3	−15.7	0.1	4.6	0.1
4	3	−14.6	0.2	9.9	0.1	3	−15.7	0.3	4.7	0.3
8	3	−14.6	0.2	9.9	0.2	3	−15.8	0.1	4.6	0.2
12	4	−14.7	0.2	10.1	0.2	3	−15.6	0.1	4.6	0.3
24	4	−14.4	0.3	10.0	0.2	3	−15.6	0.3	4.8	0.2
48	3	−14.4	0.2	10.0	0.2	3	−15.7	0.1	4.9	0.1
96	3	−14.4	0.3	9.9	0.1	3	−15.6	0.2	4.9	0.2
192	3	−14.4	0.2	9.9	0.2	3	−15.7	0.4	4.9	0.2
										
Overall		−14.5	0.3	9.9	0.2		−15.7	0.2	4.7	0.2
*F*-value		1.04		0.89			0.33		1.39	
Significance (*p*)		0.435		0.539			0.947		0.260	

*One-way ANOVA of δ^13^C and δ^15^N values among time factors for each food source. Data represent means±1 SD. Significance at p<0.05*.

**Figure 2:**
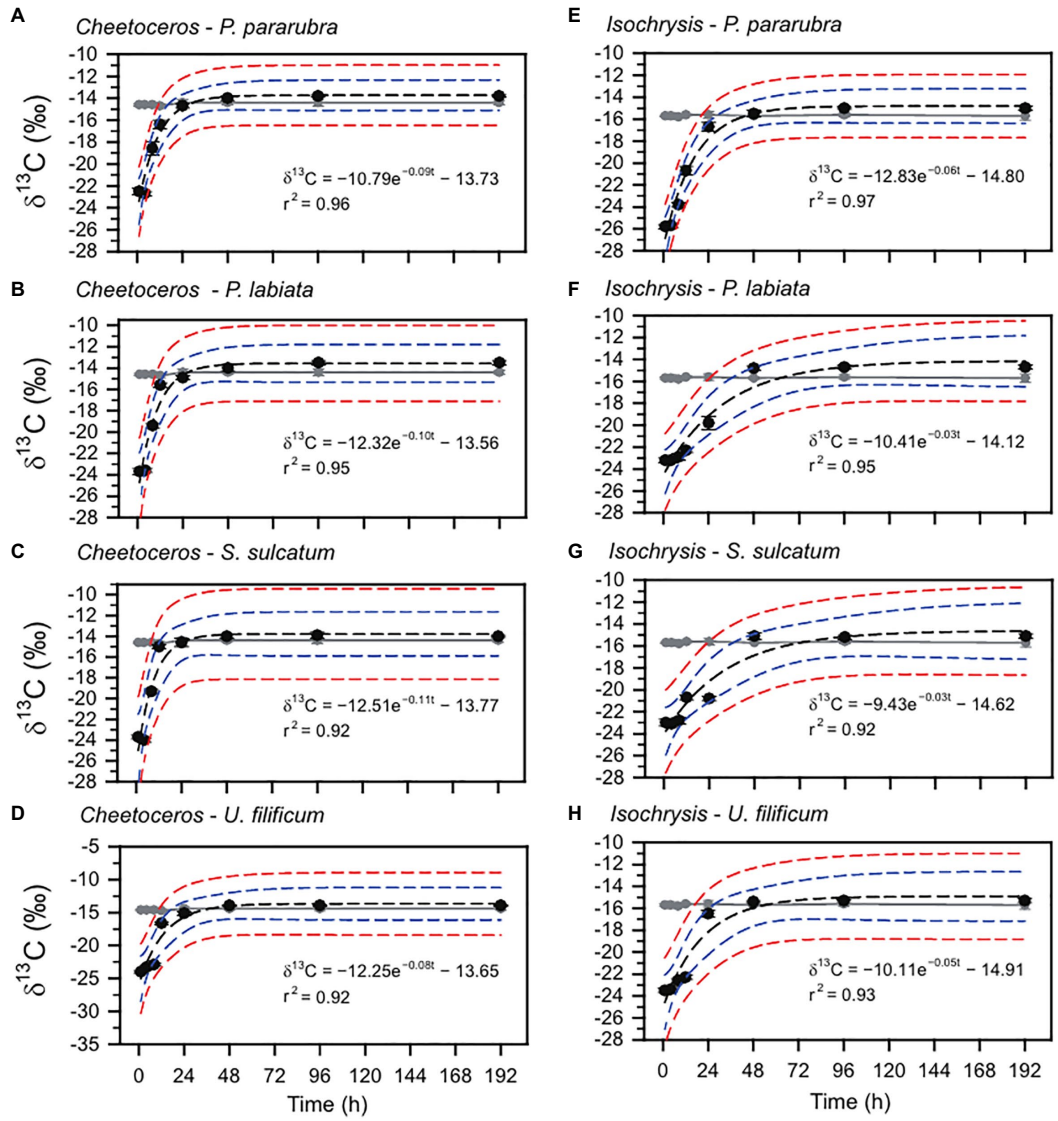
Carbon stable isotope variation and exponential model for *P. pararubra*, *Protocruzia labiate*, *Strombidium sulcatum*, and *Uronemella filificum* fed with *Chaetoceros calcitrans* (Chaeto) and *Isochrysis galbana* (Isochrysis). Black and gray circles are the mean of carbon stable isotope values (δ^13^C) for ciliates and foods, respectively. Vertical lines are ± *SD*. Black and blue dashed lines indicate the fitting curve and 95% confidence, respectively.

**Figure 3:**
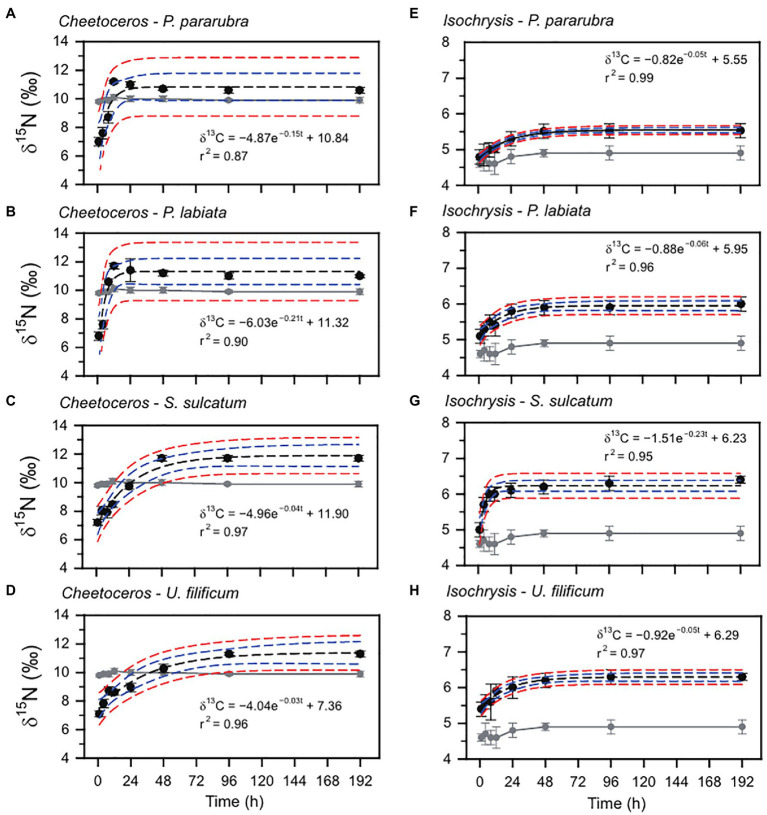
Nitrogen stable isotope variation and exponential model for *P. pararubra*, *Protocruzia labiate*, *Strombidium sulcatum*, and *Uronemella filificum* fed with *Chaetoceros calcitrans* (Chaeto) and *Isochrysis galbana* (Isochrysis). Black and gray circles are the mean of nitrogen stable isotope values (δ^15^N) for ciliates and foods, respectively. Vertical lines are ± *SD*. Black and blue dashed lines indicate fitting curve and 95% confidence, respectively.

The changes in the ciliate δ^13^C and δ^15^N values obtained from the feeding experiments are summarized in Table 2. The initial mean δ^13^C and δ^15^N values of the four ciliates were significantly different (ANOVA, *F*_3,31_=41.95, *p*<0.001 and *F*_3,31_=33.07, *p*<0.001, respectively), especially as shown by the differences between benthic (−26.2±0.2‰ and 6.2±0.2‰) and pelagic (−25.5±0.2‰ and 6.7±0.1‰) species. For all experiments, the δ^13^C and δ^15^N values of ciliates rapidly increased after the first feeding (Tukey *post-hoc* test, *p*<0.01 for all cases), which remained constant and in equilibrium with their dietary items over the next 96h (Tukey *post-hoc* test, *p*>0.05 for all cases; Figures 2, 3). Half-lives of ciliates estimated by turnover rates varied considerably, ranging from 6.1h (*S. sulcatum* fed with *I. galbana*) to 23.0h (*S. sulcatum* fed with *C. calcitrans*) for δ^13^C and from 3.1h (*S. sulcatum* fed with *I. galbana*) to 24.9h (*U. filificum* fed with *C. calcitrans*) for δ^15^N (Table 3). At equilibrium times of 96h and 192h, the differences in δ^13^C and δ^15^N values for both *C. calcitrans* (ANOVA, *F*_3,23_=11.41, *p*<0.001 and *F*_3,31_=51.84, *p*<0.001, respectively) and *I. galbana* (ANOVA, *F*_3,23_=14.50, *p*<0.001 and *F*_3,31_=33.07, *p*<0.001, respectively) were significant among the ciliates. For both the *C. calcitrans* and *I. galbana* experiments, the asymptotic δ^13^C and δ^15^N values were the highest for *P. labiata* (−13.5±0.2‰ and−14.7±0.2‰) and *S. sulcatum* (11.7±0.2‰ and 6.4±0.1‰) and the lowest for *U. filificum* (−14.0±0.2‰ and−15.3±0.1‰) and *P. pararubra* (10.6±0.2‰ and 5.5±0.1‰), respectively.

**Table 2:** Mean δ^13^C and δ^15^N values (‰) of marine ciliates (*Pseudokeronopsis pararubra*, *Protocruzia labiata*, *Strombidium sulcatum*, and *Uronemella filificum*) fed with *Chaetoceros calcitrans* and *Isochrysis galbana* during the duration of the entire experiment.

Time (h)		*Pseudokeronopsis pararubra*					*Protocruzia labiata*					*Strombidium sulcatum*					*Uronemella filificum*			
		δ^13^C		δ^15^N			δ^13^C		δ^15^N			δ^13^C		δ^15^N			δ^13^C		δ^15^N	
***Chaetoceros calcitrans***																				
	n	Mean	SD	Mean	SD	n	Mean	SD	Mean	SD	n	Mean	SD	Mean	SD	n	Mean	SD	Mean	SD
0	4	−26.3^a^	0.2	6.3^a^	0.2	4	−26.2^a^	0.2	6.1^a^	0.2	4	25.6^a^	0.2	6.7^a^	0.1	4	−25.5^a^	0.2	6.5^a^	0.2
1	5	−22.5^b^	0.3	7.0^b^	0.3	5	−23.7^b^	0.3	6.8^b^	0.3	5	−23.7^b^	0.2	7.2^b^	0.2	5	−24.0^b^	0.2	7.1^b^	0.2
4	5	−22.7^b^	0.2	7.6^b^	0.4	5	−23.6^b^	0.2	7.6^c^	0.3	5	−24.0^b^	0.2	8.0^c^	0.3	5	−23.3^c^	0.3	7.8^c^	0.3
8	5	−18.6^c^	0.6	8.7^c^	0.4	5	−19.4^c^	0.2	10.6^d^	0.2	5	−19.3^c^	0.2	7.9^c^	0.4	5	−22.9^d^	0.2	8.7^d^	0.3
12	5	−16.4^d^	0.4	11.2^e^	0.1	5	−15.6^d^	0.2	11.7^f^	0.2	5	−15.0^d^	0.2	8.5^d^	0.2	5	−16.6^e^	0.2	8.6^d^	0.2
24	5	−14.7^e^	0.2	11.0^e^	0.2	5	−14.9^e^	0.2	11.4^ef^	0.8	5	−14.6^d^	0.4	9.7^e^	0.2	5	−15.1^f^	0.3	8.9^e^	0.3
48	3	−14.0^f^	0.2	10.7^d^	0.1	3	−14.0^f^	0.3	11.2^e^	0.2	3	−14.0^e^	0.2	11.7^f^	0.2	3	−13.9^g^	0.1	10.3^f^	0.3
96	3	−13.8^f^	0.1	10.6^d^	0.2	3	−13.5^g^	0.2	11.0^e^	0.2	3	−13.9^e^	0.3	11.7^f^	0.2	3	−13.9^g^	0.1	11.3^g^	0.1
192	3	−13.8^f^	0.1	10.6^d^	0.2	3	−13.5^g^	0.2	11.0^e^	0.1	3	−14.0^e^	0.1	11.7^f^	0.2	3	−13.9^g^	0.1	11.3^g^	0.2
***Isochrysis galbana***																				
0	4	−26.2^a^	0.2	6.4^d^	0.2	4	−26.2^a^	0.2	6.0^c^	0.1	4	−25.5^a^	0.1	6.8^e^	0.1	4	−25.4^a^	0.2	6.6^e^	0.2
1	5	−25.8^b^	0.2	4.8^a^	0.2	5	−23.2^b^	0.2	5.1^a^	0.2	5	−23.0^b^	0.3	5.0^a^	0.2	5	−23.5^b^	0.2	5.4^a^	0.2
4	5	−25.7^b^	0.2	4.8^a^	0.3	5	−23.2^b^	0.2	5.3^ab^	0.2	5	−23.1^b^	0.3	5.7^b^	0.2	5	−23.4^b^	0.2	5.6^a^	0.2
8	5	−23.8^c^	0.2	5.0^ab^	0.2	5	−22.9^b^	0.3	5.5 ^b^	0.2	5	−22.8^b^	0.3	6.0^bc^	0.2	5	−22.6^c^	0.2	5.6^ab^	0.5
12	5	−20.7^d^	0.4	5.0^ab^	0.1	5	−22.3^c^	0.2	5.4^b^	0.3	5	−20.7^c^	0.2	6.0^bc^	0.2	5	−22.3^c^	0.2	5.9^b^	0.2
24	5	−16.7^e^	0.4	5.3^bc^	0.2	5	−19.8^d^	0.6	5.8^c^	0.2	5	−20.8^c^	0.2	6.1^bc^	0.2	5	−16.5^d^	0.3	6.0^b^	0.3
48	3	−15.5^f^	0.3	5.5^c^	0.2	3	−14.8^e^	0.2	5.9^c^	0.2	3	−15.1^d^	0.3	6.2^c^	0.2	3	−15.4^e^	0.1	6.2^c^	0.2
96	3	−15.0^g^	0.1	5.5^c^	0.2	3	−14.7^e^	0.2	5.9^c^	0.2	3	−15.2^d^	0.2	6.3^c^	0.2	3	−15.3^e^	0.1	6.3^c^	0.2
192	3	−15.0^g^	0.2	5.5^c^	0.2	3	−14.7^e^	0.2	6.0^c^	0.2	3	−15.1^d^	0.2	6.4^cd^	0.1	3	−15.3^e^	0.2	6.3^cd^	0.1

*Data represent means±1 SD. Means followed by the same superscript letter in each row are not significantly different (p<0.01, Tukey’s post-hoc test)*.

**Table 3:** Turnover rate and half-life (h) of carbon and nitrogen elements in marine ciliates (*Pseudokeronopsis pararubra*, *Protocruzia labiata*, *Strombidium sulcatum*, and *Uronemella filificum*) fed with Chaetoceros calcitrans and Isochrysis galbana calculated using the exponential equation (y=a+be^ct^) and regressions of carbon and nitrogen stable isotope data vs. time since diet switch.

Isotope	Diet	Species name	Turnover rate	Half-life (h)
δ^13^C	*Chaetoceros calcitrans*	*Pseudokeronopsis pararubra*	−0.09	7.4
		*Protocruzia labiata*	−0.10	6.9
		*Strombidium sulcatum*	−0.11	6.1
		*Uronemella filificum*	−0.08	9.2
	*Isochrysis galbana*	*Pseudokeronopsis pararubra*	−0.06	11.3
		*Protocruzia labiata*	−0.03	22.7
		*Strombidium sulcatum*	−0.03	23.0
		*Uronemella filificum*	−0.05	14.6
δ^15^N	*Chaetoceros calcitrans*	*Pseudokeronopsis pararubra*	−0.15	4.6
		*Protocruzia labiata*	−0.21	3.4
		*Strombidium sulcatum*	−0.04	18.2
		*Uronemella filificum*	−0.03	24.9
	*Isochrysis galbana*	*Pseudokeronopsis pararubra*	−0.05	14.7
		*Protocruzia labiata*	−0.06	11.4
		*Strombidium sulcatum*	−0.23	3.1
		*Uronemella filificum*	−0.05	13.3

### Carbon and Nitrogen Stable Isotope Fractionations

The δ^13^C and δ^15^N fractionations (Δ^13^C and Δ^15^N) for all experiments were significantly positive enrichments, with overall mean fractionations of all ciliates being 0.6±0.2 and 1.2±0.4, respectively (Table 4). There were significant differences in the Δ^13^C and Δ^15^N calculated at the initial and equilibrium (96–192h) isotope values among the ciliates (ANOVA, *F*_3,47_=26.28, *p*<0.001 and *F*_3,47_=78.67, *p*<0.001, respectively), which were relatively small. The Δ^13^C of benthic ciliates (0.64, *P. pararubra* fed with *I. galbana* to 1.00, *P. labiata* fed with *C. calcitrans*) was higher than that of pelagic ciliates (0.40, *S. sulcatum* fed with *I. galbana* to 0.57, *S. sulcatum* fed *C. calcitrans*), whereas the Δ^15^N of benthic ciliates (0.68, *P. pararubra* fed with *C. calcitrans* to 1.18, *P. labiata* fed with *I. galbana*) was relatively lower than that of pelagic ciliates (1.38, *U. filificum* fed *C. calcitrans* to 1.73, *S. sulcatum* fed *C. calcitrans*). Regardless of the dietary type, both the carbon and nitrogen isotopic fractionations of all ciliates were very similar for the same species.

**Table 4:** Trophic enrichment factors (TEFs, Δ^13^C, and Δ^15^N) of marine ciliates (*Pseudokeronopsis pararubra*, *Protocruzia labiata*, *Strombidium sulcatum*, and *Uronemella filificum*) fed with Chaetoceros calcitrans and Isochrysis galbana calculated from the equilibrium equation (Fry, 2006).

	Species name	Final value at equilibrium			TEFs
		*n*	Mean	*SD*	
δ^13^C	*Chaetoceros calcitrans* (food)	31	−14.5	0.3	
	*Pseudokeronopsis pararubra*	6	−13.8	0.1	0.69
	*Protocruzia labiata*	6	−13.5	0.2	1.00
	*Strombidium sulcatum*	6	−13.9	0.1	0.57
	*Uronemella filificum*	6	−14.0	0.2	0.51
	*Isochrysis galbana* (food)	29	−15.7	0.2	
	*Pseudokeronopsis pararubra*	6	−15.0	0.2	0.64
	*Protocruzia labiata*	6	−14.7	0.2	0.95
	*Strombidium sulcatum*	6	−15.2	0.2	0.51
	*Uronemella filificum*	6	−15.3	0.1	0.40
δ^15^N	*Chaetoceros calcitrans* (food)	31	9.9	0.2	
	*Pseudokeronopsis pararubra*	6	10.6	0.2	0.68
	*Protocruzia labiata*	6	11.0	0.1	1.06
	*Strombidium sulcatum*	6	11.3	0.1	1.38
	*Uronemella filificum*	6	11.7	0.2	1.73
	*Isochrysis galbana* (food)	29	4.7	0.2	
	*Pseudokeronopsis pararubra*	6	5.5	0.1	0.76
	*Protocruzia labiata*	6	5.9	0.1	1.18
	*Strombidium sulcatum*	6	6.3	0.1	1.59
	*Uronemella filificum*	6	6.3	0.1	1.55

## Discussion

In general, the stable isotope approach is a well-known key tool for understanding the trophic relationship between an organism and its diet. In addition, it is used to trace the pathways of organic matter within the food web, based on the common assumption of a stepwise trophic enrichment of δ^13^C by ≤1‰ (DeNiro and Epstein, 1978; Fry and Sherr, 1984) and δ^15^N by 2–4‰ (Vander Zanden and Rasmussen, 2001; Post, 2002). However, these common enrichment factors may not always apply to all individual species, because of differences in physiological status, major biochemical components (i.e., lipids, proteins, and carbohydrates), and assimilated diet during metabolic processes (DeNiro and Epstein, 1978; Focken and Becker, 1998). Furthermore, food type and quality can influence the trophic enrichment factor and isotopic turnover rates of consumer species (Remy et al., 2017). Our results showed that despite the significant differences in Δ^13^C and Δ^15^N, benthic and pelagic ciliates feeding on different microalgae had similar patterns of isotopic fractionation (i.e., TEF and isotopic turnover rate) under controlled laboratory conditions to those previously reported for other aquatic organisms (McCutchan et al., 2003; Dubois et al., 2007). Overall, the present study provides experimental quantitative data on species-specific TEFs and dietary source variations in the trophic fractionation process.

Ecological information on the influence of resource parameters on TEFs and fractionation processes of carbon and nitrogen stable isotopes in marine ciliates appears to be very uncommon compared to other aquatic organisms. In the present study, the Δ^13^C (0.4 to 1.0) and Δ^15^N (0.7 to 1.7) ciliates were found to be slightly enriched relative to their diets (both *C. calcitrans* and *I. galbana*), which was consistent with previously reported trophic fractionation ranges of aquatic consumers (Vander Zanden and Rasmussen, 2001; McCutchan et al., 2003). Similarly, previous laboratory studies on consumer TEFs have reported a general pattern showing little change in δ^13^C and relatively high enrichment in δ^15^N between prey and predators (Fry and Sherr, 1984; Minagawa and Wada, 1984). For both carbon and nitrogen, Δ^13^C and Δ^15^N were generally positive in most studies (McCutchan et al., 2003; Caut et al., 2009). However, several studies have shown that there are considerable variations in carbon and nitrogen TEFs among species due to species-specific mechanisms, the respective feeding ecology, and the associated environmental conditions (Lesage et al., 2002; Yokoyama et al., 2005; Heethoff and Scheu, 2016; Jenkins et al., 2018). Our study showed significant differences in the TEFs between benthic and pelagic ciliates, as reported by some studies on natural ecosystems, which reported that there was a clear discrimination in δ^13^C signatures between these two groups because of the difference in carbon fixation by benthic and pelagic primary producers (Fry and Sherr, 1984; France, 1995; Kang et al., 2015). Similarly, the TEFs of the marine amphipods *Ampithoe valida* and *Parhyale hawaiensis*, fed on fresh and detrital algae in laboratory experiments, showed considerable variation per species, ranging from −1.5 to −0.4 and−1.3 to −0.3 for Δ^13^C, and from −0.7 to −0.1 and 2.2 to 2.7 for Δ^15^N, respectively (Macko et al., 1982). Significant differences in Δ^13^C and Δ^15^N between herbivores (−0.4±1.1‰ and 2.5±2.5‰, respectively) and carnivores (0.9±1.0‰ and 3.2±0.4‰, respectively) were observed in laboratory and field experiments, owing to the effect of assimilate and metabolic factors by feeding type (Vander Zanden and Rasmussen, 2001). Water temperature may affect turnover rates and TEFs of carbon and nitrogen in juvenile winter flounder, which is related to differences in physiological processes (Bosley et al., 2002). Overall, despite the species-specific fractionation factors between the ciliates, their Δ^13^C and Δ^15^N did not vary with differences in species and diets under identical experimental conditions and were within the previously reported ranges.

The Δ^13^C and Δ^15^N values of animals are generally known to be influenced by different diets (McCutchan et al., 2003). Above all, herbivorous species showed highly variable Δ^15^N compared to carnivorous species based on laboratory and field estimations (Vander Zanden and Rasmussen, 2001). This tendency may be related to different food qualities, in which the protein content of different diets can affect the TEFs for consumers (McCutchan et al., 2003). Specifically, Δ^15^N may differ with the C/N ratios of diets, resulting from the degree of palatability, the consequent assimilation efficiency, and the physiological process of internal element recycling (Adams and Sterner, 2000; Remy et al., 2017). However, several studies have reported that Δ^13^C may not vary with the type and quality of diets, in contrast to the food-dependent variation in TEFs (Vanderklift and Ponsard, 2003; Mancinelli, 2012). Our study also showed no significant differences in either Δ^13^C or Δ^15^N between the two groups of ciliates fed with two different microalgal species. However, a significant difference was observed in the C/N ratios between the two diets in a laboratory experiment (unpublished data; Student’s *t*-test, *p*<0.05; 6.2±0.4 for *C. calcitrans* and 5.6±0.3 for *I. galbana*). Collectively, these results suggest that the differences in taxonomic class and quality of the two food sources for ciliates as herbivores may not result in highly variable TEFs.

Knowledge of the isotopic turnover rates of consumer tissues over time is fundamental to understanding dietary changes and quantifying the relative importance of dietary items in aquatic ecosystems (Tieszen et al., 1983; Bosley et al., 2002). As the dietary items of organisms in natural ecosystems change over time, it is necessary to retain information on the amount of time required for the organism’s isotopic ratio to equilibrate with the isotopic ratio of the last ingested food. Such information obtained through controlled laboratory experiments will improve the ecological applications of stable isotope tracers to infer the feeding strategy or history of animals (Gannes et al., 1997; Herzka et al., 2001). In the present study, the turnover time for both benthic and pelagic ciliates to equilibrate with the isotopic ratios of the two microalgae as their dietary items was 96h. Despite the substantial variability in half-lives among ciliates (6.1–23.0h for δ^13^C and 3.1–24.9h for δ^15^N), the turnover periods were relatively very fast in comparison with other animals, showing a wide range from several days to 1year (Bosley, 1998). Given that the turnover rate of the amphipod *Gammarus* for δ^13^C (half-life 12.5 d and 51.6 d fed with animal and litter, respectively) was fast because of the rapid assimilation of food sources (Remy et al., 2017), our results may be unique to the physiological responses of ciliates. Several studies have demonstrated that the turnover rates of organisms are very specific to the taxon or species level (Fry and Arnold, 1982; Tieszen et al., 1983; Yokoyama et al., 2005). Hobson and Clark (1992) found that the turnover rates of Japanese quail may vary according to the life stage and the particular tissue and body compartment, owing to differences in the level of metabolic activity.

The turnover rate of an organism can be significantly influenced by the speed of growth, likely reflecting the rapid shift of the isotopic ratios of consumer tissues to the existing diet (Bosley et al., 2002). By comparing rapidly and slowly growing individuals, Fry and Arnold (1982) demonstrated that turnover rates of post-larval brown shrimp can be directly related to growth. In fact, the rapid change in the carbon and nitrogen isotopic ratios of the four ciliates may be closely associated with their fast growth and metabolism; similarly, high growth rates are observed in microalgal species (Banse, 1982). Therefore, our results suggest that the rapid assimilation of microalgae, together with the exponential growth of ciliates, may contribute to their fast turnover rates.

## Conclusion

The results of this study, involving controlled feeding experiments with microalgal diets, indicate that the TEFs of carbon and nitrogen for marine ciliates are similar to those found for common marine organisms with very little food-dependent variation. Although stable isotope analysis is a useful tool for assessing the trophic role of an organism in food webs, isotopic fractionation is generally species-specific in animals, and information on the TEFs of carbon and nitrogen for marine ciliates is still lacking. In this respect, the knowledge of species-specific TEFs needs to be based on precise estimation of the trophic level of an organism and the contribution of potential food sources to nutrition, by mixing models. Furthermore, considering the trophic importance of marine ciliates with respect to carbon flow, our study, which quantified their specific isotopic fractionation, validates the importance of stable isotope ecology in marine microbial food webs. Overall, these results are expected to provide fundamental information on the trophic transfer of carbon, nitrogen, and energy flow through microbial pathways in marine ecosystems.

## Data Availability Statement

The original contributions presented in the study are included in the article/supplementary material; further inquiries can be directed to the corresponding author.

## Author Contributions

JYP and HJP designed the experiments and wrote the main manuscript text. J-HJ and HGP provided all the ciliate and microalgae samples. JHK performed the statistical analyses. C-KK revised the manuscript. All authors analyzed the data and reviewed the manuscript.

## Funding

This research was supported by the National Research Foundation of Korea (NRF-2018R1C1B6004501), Ministry of Science and ICT, and Long-term change of structure and function in marine ecosystems of Korea project funded by the Ministry of Oceans and Fisheries, Korea.

## Conflict of Interest

The authors declare that the research was conducted in the absence of any commercial or financial relationships that could be construed as a potential conflict of interest.

## Publisher’s Note

All claims expressed in this article are solely those of the authors and do not necessarily represent those of their affiliated organizations, or those of the publisher, the editors and the reviewers. Any product that may be evaluated in this article, or claim that may be made by its manufacturer, is not guaranteed or endorsed by the publisher.
